# P44 Optimizing blood culture pathway and antibiotics stewardship through co-design interventions

**DOI:** 10.1093/jacamr/dlaf118.051

**Published:** 2025-07-14

**Authors:** Alison Prendiville, Wenbo Ai, Deborah Bamber, Carolyn Tarrant

**Affiliations:** London College of Communication, University of the Arts London, London, SE1 6SB, UK; London College of Communication, University of the Arts London, London, SE1 6SB, UK; Department of Population Health Sciences, University of Leicester, Leicester, LE1 7RH, UK; Department of Population Health Sciences, University of Leicester, Leicester, LE1 7RH, UK

## Abstract

**Background:**

Blood culture sampling is important for clinical decision making so that patients can receive the best infection specific antibiotic at the earliest stage of their treatment, and to reduce antibiotic overuse. The i Sample project is an NIHR-funded research study that aims to create a bundle of design interventions to optimize blood culture sampling for patients with suspected severe infection in acute care in England. The study involves an embedded co-design process. This poster will report on the initial stages of the co-design process and the visual resources developed to date and will provide an overview of the use of co-design methodology to develop interventions to optimize blood culture sampling.

**Methods:**

Our primary design methods involve design ethnography in Stage 1 and co-design workshops to develop interventions in Stage 2 within three NHS trusts in England. In Stage 1, initial observations have been conducted to document staff roles in requesting and taking blood samples, and to inform mapping of the blood sampling pathway. Participatory observation notes were documented incorporating a variety of formats such as diary, drawings, storyboard, text, and quotations. Analysis of observational data identifies gaps in the process, and how blood culture sampling is connected to antibiotic prescribing and clinical decision-making. This initial stage has informed the development of visual materials to support co-design workshops in Stage 2.

**Results:**

A co-discovery poster, service flow map, and prompt question cards were developed to facilitate the initial workshop, serving as a communication tool to stimulate discussion and understand the pathway and key points for intervention. Visual mapping of blood culture pathways is vital for the development of interventions that support a holistic, systems wide approach to optimizing blood culture sampling. These visual tools represent actors, physical environments, prioritization, and challenges in existing practices for blood culture sampling for acute medical patients. Various visual design materials will be developed, selected, and used in a series of four co-design workshops at each of the hospital sites. Examples of envisioned visual materials: stakeholder map, a blood culture visual timeline, patient and healthcare staff journey maps, storyboards.

**Conclusions:**

Co-design methods will be used to iteratively develop interventions that target behavioural factors, amplify collective efforts for all stakeholders, and address organizational and system issues in terms of blood culture sampling and antimicrobial stewardship. The co-design methods will be selected based on the stage of the workshops and the type of interventions. For example, participatory group discussion, co-developing personas and storyboards, sketching and prototyping, role-playing and brainstorming with cards, design scenarios. A final workshop with stakeholders from across England will enable to development of generalizable learning from this co-design work.

